# More widespread functionality of posterior language area in patients with brain tumors

**DOI:** 10.1002/hbm.26801

**Published:** 2024-08-01

**Authors:** Riho Nakajima, Takahiro Osada, Masashi Kinoshita, Akitoshi Ogawa, Hirokazu Okita, Seiki Konishi, Mitsutoshi Nakada

**Affiliations:** ^1^ Department of Occupational Therapy, Faculty of Health Science, Institute of Medical, Pharmaceutical and Health Sciences Kanazawa University Kanazawa Japan; ^2^ Department of Neurophysiology Juntendo University School of Medicine Tokyo Japan; ^3^ Department of Neurosurgery, Faculty of Medicine, Institute of Medical, Pharmaceutical and Health Sciences Kanazawa University Kanazawa Japan; ^4^ Department of Physical Medicine and Rehabilitation Kanazawa University Hospital Kanazawa Japan; ^5^ Sapiens Life Sciences Evolution and Medicine Research Center Kanazawa University Kanazawa Japan

**Keywords:** direct electrical stimulation, glioma, posterior language area, reorganization, resting‐state functional magnetic resonance imaging, Wernicke's area

## Abstract

Damage to the posterior language area (PLA), or Wernicke's area causes cortical reorganization in the corresponding regions of the contralateral hemisphere. However, the details of reorganization within the ipsilateral hemisphere are not fully understood. In this context, direct electrical stimulation during awake surgery can provide valuable opportunities to investigate neuromodulation of the human brain in vivo, which is difficult through the non‐invasive approaches. Thus, in this study, we aimed to investigate the characteristics of the cortical reorganization of the PLA within the ipsilateral hemisphere. Sixty‐two patients with left hemispheric gliomas were divided into groups depending on whether the lesion extended to the PLA. All patients underwent direct cortical stimulation with a picture‐naming task. We further performed functional connectivity analyses using resting‐state functional magnetic resonance imaging (MRI) in a subset of patients and calculated betweenness centrality, an index of the network importance of brain areas. During direct cortical stimulation, the regions showing positive (impaired) responses in the non‐PLA group were localized mainly in the posterior superior temporal gyrus (pSTG), whereas those in the PLA group were widely distributed from the pSTG to the posterior supramarginal gyrus (pSMG). Notably, the percentage of positive responses in the pSMG was significantly higher in the PLA group (47%) than in the non‐PLA group (8%). In network analyses of functional connectivity, the pSMG was identified as a hub region with high betweenness centrality in both the groups. These findings suggest that the language area can spread beyond the PLA to the pSMG, a hub region, in patients with lesion progression to the pSTG. The change in the pattern of the language area may be a compensatory mechanism to maintain efficient brain networks.

## Graphical abstract

Reorganization of language area in the ipsilateral hemisphere was analyzed combined using direct electrical stimulation and resting‐state functional MRI. Accordingly, language area could be spread to the posterior supramarginal gyrus beyond the original language area under the condition of lesion progression to the posterior superior temporal gyrus, a hub region.

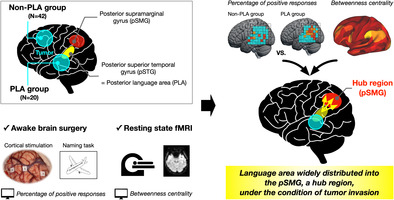

**Practitioner Points**

- We studied characteristics of reorganization of the posterior language area using direct electrical stimulation and resting‐state functional MRI.
- The change in the pattern of the language area can occur under the condition of tumor invasion to the posterior language area.
- The posterior language area can spread into the posterior supramarginal gyrus, a hub region, beyond the original area, and the mechanism is considered to be a compensatory way to maintain efficient brain networks.

## 1 INTRODUCTION

The reorganization process is a mechanism to prevent or recover neurological and cognitive deficits by reallocating brain function outside a lesioned area (Cargnelutti et al., 2020; Duffau, 2014). Language areas are frequently rearranged at the cortical level, especially with chronic lesions such as tumors that grow over time; this can lead to large‐scale reorganization of the neural network (Ng et al., 2023; Saito et al., 2016; Southwell et al., 2016). Indeed, preoperative language ability has often been found to be normal in patients with glioma despite the presence of a tumor in the language areas, presumably due to preoperative reorganization (Piai et al., 2020; Saito et al., 2020).

Reorganization can occur in the following patterns: compensation in the contralateral hemisphere, in the ipsilateral hemisphere distant from the lesion, and in the ipsilateral hemisphere surrounding the lesion (Cargnelutti et al., 2020; Nieberlein et al., 2023). In the posterior language area (PLA; i.e., Wernicke's area in the left posterior part of the superior temporal gyrus [pSTG]), compensation in the contralateral hemisphere has been reported in neuroimaging studies (Balter et al., 2019; Mbwana et al., 2009; Partovi et al., 2012; Stockert et al., 2020). There are also fewer reports on reorganization in distant areas in the ipsilateral hemisphere (Avramescu‐Murphy et al., 2017; Mbwana et al., 2009; Stockert et al., 2020). On the other hand, it is difficult to detect compensation in peri‐lesion areas in the ipsilateral hemisphere (Piccirilli et al., 2023; Stockert et al., 2020), presumably because of the difficulty of neuroimaging analyses in estimating the spatial extent of brain activity (Enatsu et al., 2013; Sarubbo et al., 2012). Another reason why perilesional reorganization is less likely to be detected is that it is an early compensatory mechanism (Nieberlein et al., 2023), and it may be difficult to detect in acute onset diseases such as stroke.

In contrast to numerous non‐invasive methods for investigating brain reorganization, brain surgery sometimes provides more invasive research opportunities to measure neural activity or intervene in brain mechanisms. Among invasive methods, cortical stimulation using direct electrical stimulation (DES) during awake brain surgery for epilepsy or glioma has the advantage of higher spatial resolution and greater sensitivity in detecting reorganization (Enatsu et al., 2013; Ng et al., 2023; Pallud et al., 2017; Sarubbo et al., 2012). We have previously demonstrated that the homunculus of motor function was changed by reorganization within the same brain gyrus using DES (Nakajima et al., 2020). As a complementary approach, resting‐state functional magnetic resonance imaging (rsfMRI) provides valuable insights into neural circuits associated with cognitive functions at the whole‐brain level (Fox & Raichle, 2007). In network analyses using graph theory, certain regions that interact with many other regions are considered critical brain areas within the network, commonly referred to as hubs (Bullmore & Sporns, 2009; Crossley et al., 2014). We previously reported that regions with high betweenness centrality (BC), an index of network importance based on functional connectivity at the whole‐brain level, played a critical behavioral role in brain networks (Fujimoto et al., 2020). In this context, we defined hubs as regions with high centrality, estimated by graph‐theoretical analysis (Sporns et al., 2007; Tanglay et al., 2023).

Recent studies showed reorganizations in the functional networks of the brain in patients with both low‐ and high‐grade gliomas (Semmel et al., 2021). Therefore, it is plausible that brain tumors induce a spatial shift in hub regions as well (Tuovinen et al., 2016). The preservation of the hub region is an important factor in postoperative functional recovery (Lee et al., 2020). However, the relationship between the cortical reorganization induced by brain tumors and the way brain networks change, specifically with regard to the role of hub regions, is not well understood.

**FIGURE 1:**
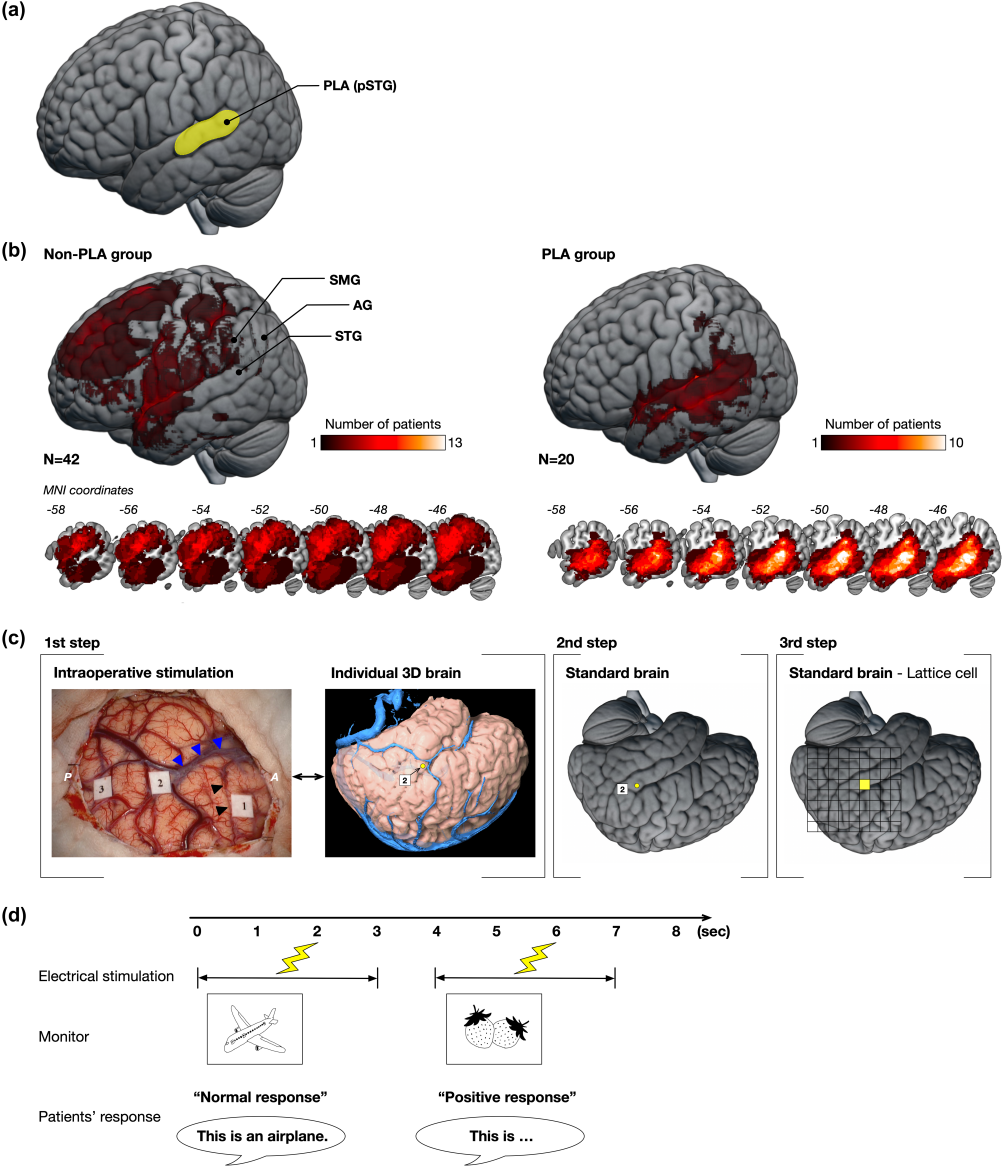
Methods of this study. (a) The location of the posterior language area (PLA), which corresponds to the posterior part of the superior temporal gyrus (pSTG). (b) Maps of tumor overlap across patients in the non‐PLA and PLA groups. AG, angular gyrus; SMG, supramarginal gyrus; STG, superior temporal gyrus. (c) The schema for identifying the spatial location of positive responses. First step: Positive points can be identified by number tag labels on the cortical surface via intraoperative video records. Each location with a positive response is plotted on the individual 3D brain using iPlan Stereotaxy 3.0 software (BrainLab). Blue triangle, Sylvian fissure; black triangle, central sulcus; P, posterior; A, anterior. Second step: Every positive point is transferred to the standard brain. Third step: For statistical analysis, the standardized positive points are mapped onto the corresponding lattice cell. (d) The schema of the picture‐naming task during awake surgery. Patients are instructed to name the pictures, which are presented on the monitor, in Japanese. We defined the patients' answers as a “positive response” or “normal response” according to their response.

In this study, we combined DES during awake surgery and preoperative rsfMRI to characterize cortical reorganization after a tumor lesion in the ipsilateral hemisphere. Sixty‐two patients with left hemispheric gliomas (Figure S1) were divided into two groups, namely the PLA and non‐PLA groups, based on whether the lesion extended to the PLA (Figure 1a,b, Table 1). Patients performed a picture‐naming task during cortical stimulation, and positive (impaired) and normal (unimpaired) responses were mapped on the brain surface within and surrounding the PLA (Figure 1c,d). We further conducted rsfMRI in a subset of patients and performed network analyses of functional connectivity to examine how cortical organization is rearranged in patients with tumor invasion.

**TABLE 1:** Demographic and clinical characteristics of participants.

Characteristics	Value		
	Non‐PLA group (*n* = 42)	PLA group (*n* = 20)	*p*‐Value
Age			.19
Mean ± SD	48.5 ± 12.6	43.9 ± 13.8	
Range	17–72	16–72	
Sex			.66
Male	27	14	
Female	15	6	
Tumor location			<.0001
Frontal	25	0	
Parietal	9	4	
Temporal	8	16	
WHO grade			.26
2	18	6	
3	12	4	
4	12	10	
Preoperative tumor volume (cm^3^)			.96
Mean ± SD	35.5 ± 35.9	36.0 ± 35.7	
Amplitude of direct electrical stimulation (mA)			.32
Mean ± SD	4.0 ± 1.1	4.3 ± 1.3	
Range	2.5–6	3–6	

*Note*: Each value was compared using *t*‐test or chi‐square test.

Abbreviations: PLA, posterior language area; SD, standard deviation.

## 2 MATERIALS AND METHODS

### 2.1 Participants

A total of 137 patients underwent awake surgery for resection of World Health Organization grades 2, 3, and 4 gliomas at Kanazawa University Hospital between May 2014 and February 2021. In accordance with the inclusion criteria, the following patients were excluded (Figure S1): those with lesions on the right side (*n* = 57), unsuccessful awake monitoring due to insufficient wakefulness and severe aphasia (*n* = 4), unsuccessful recording (*n* = 3), and no examination of the language area and its surrounding areas (*n* = 11). Consequently, 62 patients met our inclusion criteria (mean age ± standard deviation: 47.0 ± 13.1 years). To assess language function before surgery, we used a picture‐naming task of high‐frequency words that consisted of 60 pictures. Most patients (*n* = 60) had normal language function before surgery. A few patients (*n* = 2) had very mild aphasia, but this did not affect their ability to perform the picture‐naming task during awake surgery.

Patients were divided into the PLA (*n* = 20) and non‐PLA (*n* = 42) groups based on tumor location (Figure S2A). The concept of the PLA, or Wernicke's area is currently not definitively established, and various areas are defined subjectively (Tremblay & Dick, 2016). We defined the PLA as the posterior portion of Brodmann's area 22, synonymous with Wernicke's area in the left pSTG (Figure 1a), as it has been classically defined (Mesulam et al., 2015; Tremblay & Dick, 2016). The PLA group included patients in whom the preoperative tumor overlapped with the posterior portion of Brodmann's area 22, and the other patients without this feature were included in the non‐PLA group. Tumor location was determined based on a hyperintense lesion on fluid‐attenuated inversion recovery (FLAIR) MRI for grades 2 and 3 gliomas and gadolinium enhancement for grade 4 gliomas. The demographic and clinical characteristics of each group are summarized in Table 1. There were no significant differences between the non‐PLA and PLA groups except in tumor location.

### 2.2 MRI‐based group classification

All patients underwent preoperative structural MRI, including T1‐weighted and FLAIR images, as part of the standard care (typically, T1‐weighted images: repetition time [TR], 6.04 ms; echo time [TE], 2.4 ms; voxel size, 0.94 × 0.94 × 1.00 mm; field of view [FOV], 240 × 240 × 180 mm; FLAIR images: TR, 6000 ms; TE, 114.5 ms; voxel size, 0.70 × 0.47 × 0.47 mm; FOV, 180 × 240 × 240 mm). MRI was performed using a 3.0‐T MR scanner (Signa Excite HDx 3.0 T [GE Healthcare, Little Chalfont, UK] or Philips Ingenia [Philips Healthcare, Best, the Netherlands]). T1‐weighted and FLAIR images were spatially normalized to the Montreal Neurological Institute (MNI) template with a nonlinear transformation using SPM12 (www.fil.ion.ucl.ac.uk/spm/). This procedure employs unified models implemented in SPM12, which enables the normalization of images, even in the presence of lesions, to the MNI space by performing tissue matching based on the tissue probability map template, including gray matter, white matter, and cerebrospinal fluid (Ashburner & Friston, 2005). The normalized structural images were resampled with a resolution of 1.0‐mm isotropic voxel size. The accuracy of the transformation was verified by comparing anatomical landmarks such as the sulci and brainstem. The preoperative tumor was manually contoured and transformed into binarized images using MRIcron software (people.cas.sc.edu/rorden/mricron/) (Brett et al., 2001). Each reconstruction was initially performed by the first author (R.N.) and systematically reviewed by a neurosurgeon (M.K.). We examined whether the binarized images of the tumors overlapped with Brodmann's areas 22 and divided the patients into the PLA and non‐PLA groups. In accordance with a previous study, the PLA group was defined as patients in whom Brodmann's area 22 was involved by at least 50 voxels (Biesbroek et al., 2013). The mean number of overlapping voxels was 1946 ± 2004. The maximum across‐subject overlap was in the deep part of the precentral gyrus for the non‐PLA group and in the pSTG and middle temporal gyrus for the PLA group (Figure 1b).

### 2.3 Spatial topography of cortical stimulations

Each patient underwent surgical resection of the brain tumor with DES while awake, according to the guidelines of the Awake Craniotomy Guidelines Committee of the Japan Awake Surgery Conference (Kayama, 2012). Surgery was performed using the asleep‐awake‐asleep technique, as previously described (Gogos et al., 2020). Cortical stimulation with the language task was performed before resection. Electrical stimulation using a bipolar electrode with 5‐mm‐spaced tips delivering a biphasic current (pulse frequency of 60 Hz, pulse duration of 0.2 ms, stimulus duration of 3 s, amplitude of 1.5–6 mA, Nihon Kohden Neuromaster; Nihon Kohden, Tokyo, Japan) was used to determine language areas. The stimulus current is determined in accordance with a previously described method (Gogos et al., 2020). The mean amplitudes of DES for the non‐PLA and PLA groups were 4.0 ± 1.1 mA and 4.3 ± 1.3 mA, respectively (*p* = .50, *t*‐test), which were mostly in accordance with a previous report (Hervey‐Jumper et al., 2015).

Each stimulation point of DES was identified using a numbered tag placed on the cortical surface (Figure 1c, first step). The locations of all DESs were identified using a video‐recorded bipolar electrode tip. These anatomical locations were identified using the following steps, as previously reported (Lu et al., 2021; Sarubbo et al., 2020). First, all points were plotted on individual 3D T1‐weighted images using iPlan Stereotaxy 3.0 software (BrainLab, München, Germany). Anatomical landmarks (i.e., gyri, sulci, and vessels) and surgical reports were used to determine the optimal location of electrical stimulation responses (Tate et al., 2014). These plotted points on the 3D brain were then automatically plotted on 2D‐MRI. Thereafter, the mapping points of the original 2D‐MRI were transferred to normalized individual MRI, considering the anatomical structure. Next, each point was plotted on the brain surface of a normalized 3D MNI template using MRIcroGL software (github.com/rordenlab/MRIcroGL/) (Figure 1c, second step). Positive points displayed on the MNI template were double‐checked to ensure that the anatomical location was the same as that on the original 3D brain by two independent neurosurgeons (MK and MN). The location of each point was then converted to square sections on a lattice (size, 10 × 10 mm) covering the PLA (Figure 1c, third step) (Southwell et al., 2016). We then calculated the ratio of the number of patients with positive responses to the number of electrically stimulated patients for each square section. If a patient had two or more positive points in a square section, that square was counted as only one positive response (Figure S2B). The site of stimulation differed across patients depending on the location of the tumor and the extent of craniotomy. The same was applicable for normal responses. Finally, the accuracy of the anatomical location of the square section was verified by comparison with the individual T1‐weighted image. All procedures were performed by the first author (R.N.) and carefully validated by neurosurgeons (M.K. and M.N.).

### 2.4 Intraoperative assessments of language function

For intraoperative language mapping with DES, the patients were asked to perform a picture‐naming task identical to the preoperative assessment (Figure 1d). The pictures were presented on a monitor using PowerPoint (Microsoft, Redmond, WA). We defined incorrect responses as “positive responses” and correct responses as “normal responses.” The number of stimulations in each region was determined based on clinical considerations, such as tumor location and surgical planning, and differed for each patient. Therefore, the total number of stimulations differed in every region. Each region was stimulated a maximum of three times, and if a positive response was found at least twice, the region was defined as a positive point (Hervey‐Jumper et al., 2015). Otherwise, the region was defined as a normal point. The types of positive responses in the naming task included anomia, perseveration, phonemic aphasia, repetition disorder, and semantic aphasia (Leonard et al., 2019; Sarubbo et al., 2020). All brain regions that had positive responses were not resected to prevent postoperative aphasia. DES of the same region was not repeated more than three times to avoid intraoperative seizures. All intraoperative assessments were performed by a trained occupational therapist (R.N.) and a speech therapist (H.O.). After surgery, the error type classification in the picture‐naming task was confirmed by a senior neurosurgeon who was blinded to the patient group using intraoperative video recordings. The ratio of the number of patients with positive responses to the number of patients stimulated was calculated in each square section and compared between the PLA and non‐PLA groups using the chi‐squared test. All statistical analyses were performed using JMP Pro version 16.2.0 (SAS Institute Japan, Tokyo, Japan).

### 2.5 rsfMRI

In total, 40 patients, including 26 patients in the non‐PLA group (mean age: 48.5 ± 12.4 years; 16 males, 10 females) and 14 in the PLA group (mean age: 43.6 ± 13.4 years; 10 males, 4 females), underwent rsfMRI before surgery (Figure S2A). The demographic and clinical characteristics of these patients are summarized in Table 2. To compare the results of rsfMRI with a control group, we recruited 22 patients with age‐matched right cerebral hemispheric gliomas (mean age: 45.5 ± 16.2 years; 14 males, 8 females; WHO grade 2, *n* = 12; WHO grade 3, *n* = 10). Moreover, we utilized 40 subjects from the Human Connectome Project (HCP) as the normal control group (31–35 years: *n* = 27, 12 males and 15 females; 36–40 years: *n* = 13, 4 males and 9 females).

**TABLE 2:** Demographic and clinical characteristics of participants who underwent rsfMRI.

Characteristics	Value		
	Non‐PLA group (*n* = 26)	PLA group (*n* = 14)	*p*‐Value
Age			.26
Mean ± SD	48.5 ± 2.5	43.6 ± 3.4	
Range	24 to 72	19 to 72	
Sex			.53
Male	16	10	
Female	10	4	
Tumor location			<.0001
Frontal	15	0	
Parietal	7	3	
Temporal	4	11	
WHO grade			.58
2	11	5	
3	8	3	
4	7	6	
Preoperative tumor volume (cm^3^)			.96
Mean ± SD	30.3 ± 30.3	36.2 ± 38.9	

*Note*: Each value was compared using *t*‐test or chi‐square test.

Abbreviations: PLA, posterior language area; SD, standard deviation.

Functional rsfMRI was obtained using either single‐ or multi‐band gradient‐echo echo‐planar imaging (EPI) sequences. For single‐band EPI, the acquisition parameters were as follows: TR, 2.5 s; TE, 30 ms; voxel size, 3.3 × 3.3 × 3.2 mm; matrix size, 64 × 64; 40 slices with a 0.8‐mm gap; and number of volumes, 240 (total scan time, 10 min). For multi‐band EPI, the acquisition parameters were as follows: TR, 1.5 s; TE, 30 ms; voxel size, 2.4 × 2.4 × 2.4 mm; matrix size, 96 × 96; 54 contiguous slices; multi‐band factor, 3; and number of volumes, 300 (total scan time, 7.5 min). During resting‐state scans, the patients were instructed to keep their eyes open.

rsfMRI was preprocessed using SPM12, FSL (fsl.fmrib.ox.ac.uk/fsl/fslwiki/) (Smith et al., 2004), and the HCP pipelines (Glasser et al., 2013; Glasser et al., 2016), as previously described (Nakajima et al., 2022; Osada et al., 2021). The preprocessing steps included slice‐timing correction, realignment, and spatial normalization to the MNI template with interpolation to a 2.0 × 2.0 × 2.0‐mm space. Spatial normalization was performed nonlinearly based on the unified models implemented in SPM12. Time‐series data were cleaned using the ICA‐FIX method (Salimi‐Khorshidi et al., 2014). Image data were projected onto the 32 k fs_LR surface space using MSMSulc (Glasser et al., 2016) and Ciftify (Dickie et al., 2019). Furthermore, the mean cortical grayordinate signal was regressed from the time‐series data, followed by spatial smoothing with a full width at half maximum of 6 mm. In the present study, global signal regression (i.e., mean cortical grayordinate signal regression) was employed to improve the specificity of functional connectivity analysis (Fox et al., 2005; Murphy & Fox, 2017). While the use of global signal regression is highly controversial, previous research reported that global signal regression increased the test–retest reliability of graph theoretical measures (Braun et al., 2012). Other studies showed opposite results (Liang et al., 2012).

We calculated functional connectivity and BC among the 360 cerebrocortical parcels (i.e., functional areas) available from the HCP (Glasser et al., 2016). The preprocessed rsfMRI data were averaged across the vertices in each parcel. Temporal correlation coefficients between the cerebrocortical parcels were calculated and transformed to Fisher's z‐values for functional connectivity. A proportional threshold of the top 10% was applied to create a binary, undirected resting‐state cerebral network. The Brain Connectivity Toolbox (https://sites.google.com/site/bctnet/) (Rubinov & Sporns, 2010) was used to define the cerebral network and calculate the BC, which measures the proportion of the shortest paths between all parcel pairs in the cerebral network (Fujimoto et al., 2020, 2022). The BC of parcel *i* was calculated as the proportion of the shortest paths between parcels *j* and *h* that passed through parcel *i*.

(1) $$b_{i}=\frac{1}{\left(N-1\right)\left(N-2\right)}\sum_{h\neq i,h\neq j,j\neq i}\frac{\rho_{\mathrm{hj}}\left(i\right)}{\rho_{\mathrm{hj}}},$$

where *ρ*_*hj*_(*i*) is the number of shortest paths between parcels *h* and *j* passing through parcel *i*, *ρ*_*hj*_ is the number of shortest paths between parcels *h* and *j*, and *N* is the number of parcels. A parcel with high BC mediates a high proportion of information traffic if the information is conveyed in the cerebral network along the shortest path. Such parcels may control the information passage or act as bottlenecks for information flow.

## 3 RESULTS

### 3.1 Intraoperative findings

**FIGURE 2:**
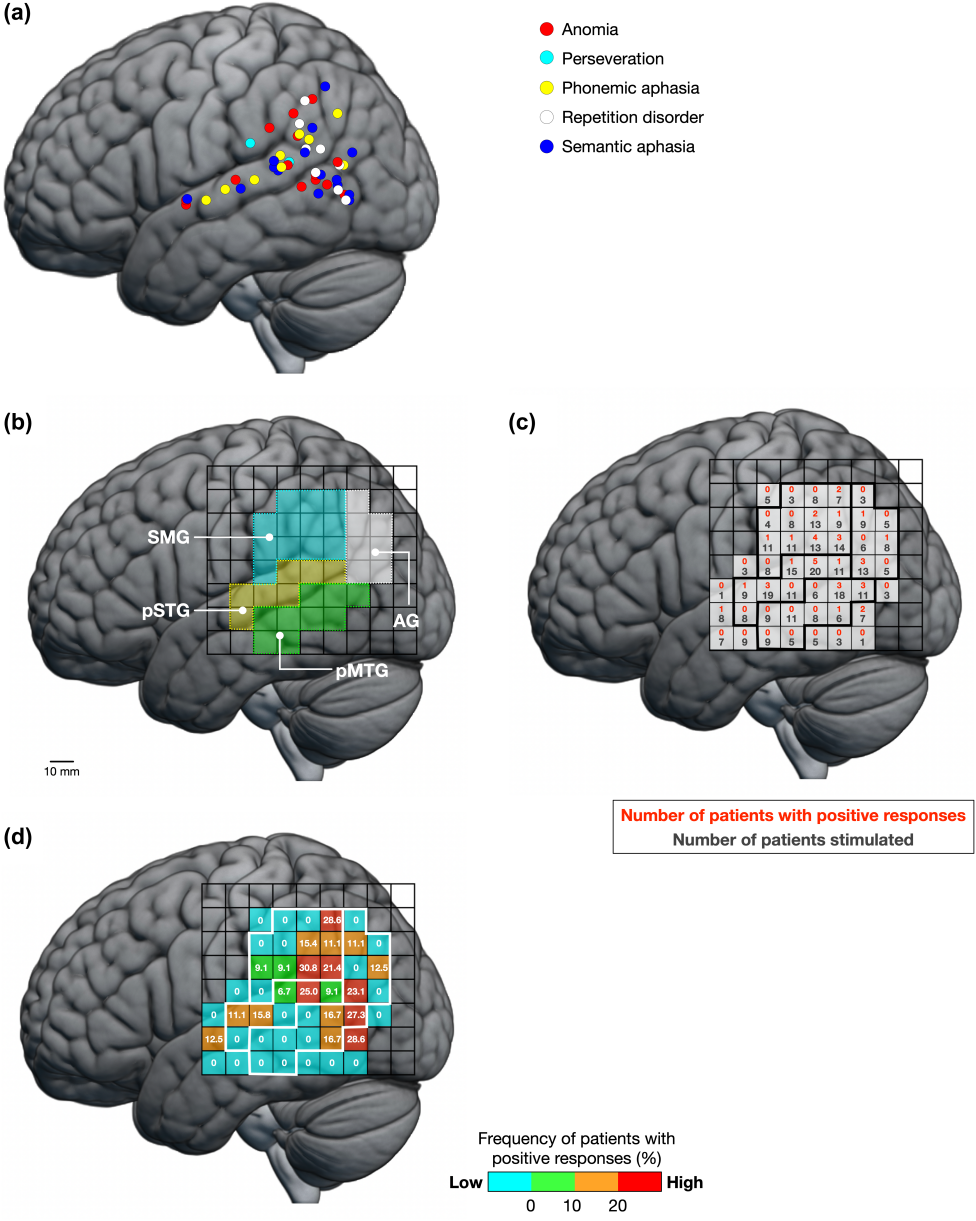
Positive points for all patients. (a) Each error type is represented in the following color: Red, anomia; cyan, perseveration; yellow, phonemic aphasia; white, repetition disorder; blue, semantic aphasia. (b) The posterior language area is divided into four areas based on anatomical structures: Posterior part of the superior temporal gyrus (pSTG), posterior middle temporal gyrus (pMTG), supramarginal gyrus (SMG), and angular gyrus (AG). (c) Raw data for positive responses in all patients. In each cell, the lower black letters indicate the number of electrically stimulated patients, whereas the upper red letters indicate the number of patients with positive responses. The stimulation points differ in each patient depending on the location of the tumor and the extent of craniotomy. Therefore, the number of electrically stimulated patients (black letters) per square is different from the total number of patients in each group. (d) The frequency of positive responses is calculated as the ratio (%) of the number of patients with positive responses to the number of patients stimulated. Areas with a high frequency of patients with positive responses are indicated by warm colors, whereas areas with a low frequency of patients with positive responses are indicated by cool colors. Each side of the square is 10 mm.

A total of 48 positive points and 488 normal points were observed during the picture‐naming task with DES. Error responses for the positive points included anomia (14 points), perseveration (2 points), phonemic aphasia (9 points), repetition disorder (8 points), and semantic aphasia (15 points) (Figure 2a). The PLA and its surrounding areas were classified into the pSTG, posterior middle temporal gyrus (pMTG), supramarginal gyrus (SMG), and angular gyrus (AG) according to their anatomical structure (Figure 2b). Positive points were distributed around the temporoparietal junction, including the pSTG, pMTG, SMG, and AG. Details of the MNI coordinates are summarized in Supplementary Table S1. Removal of areas with any type of positive response was considered to cause postoperative aphasia; such areas were not resected during surgery. In accordance with a previous study (Saito et al., 2020), all types of response errors were treated as naming errors in the subsequent analysis. Figure 2c,d show the ratio of the number of patients with positive responses to the number of electrically stimulated patients for each square section. Areas with a high proportion of positive responses were clustered from the pSTG to the SMG. Moreover, there were areas with a high proportion of positive responses in the pMTG and AG.

**FIGURE 3:**
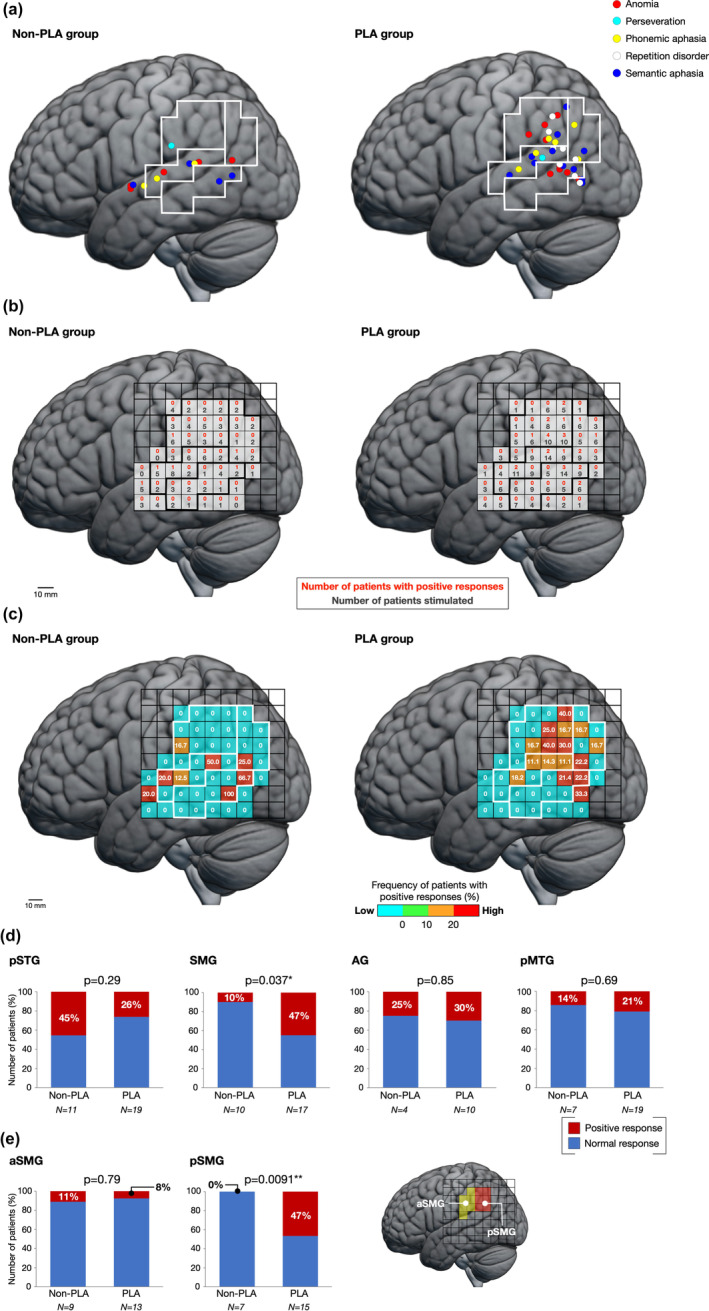
Intraoperative findings in two groups. (a) Positive points in the non‐posterior language area (PLA) and PLA groups. (b) Raw data for positive responses in the non‐PLA and PLA groups. The lower black letters indicate the number of electrically stimulated patients, and the upper red letters indicate the number of patients with positive responses. (c) The frequency of positive responses in each group. (d) The percentages of positive responses in the four areas in the non‐PLA and PLA groups. Only the supramarginal gyrus (SMG) is found to be significantly different between the groups. The number under the horizontal axis represents the number of patients stimulated in each gyrus. Red, positive response; blue, normal response. (e) The SMG is divided into two parts, anterior and posterior SMG (anterior part of the supramarginal gyrus [aSMG] and posterior part of the supramarginal gyrus [pSMG]). *p < .05; **p < .01.

The patients were divided into two groups based on tumor location. In the non‐PLA group, 20 patients were stimulated at the PLA and the surrounding area. In total, 13 positive and 226 normal points were observed in the non‐PLA group (Figures 3a and S3A). Error responses for the positive points included anomia (5 points), perseveration (1 point), phonemic aphasia (3 points), and semantic aphasia (4 points). In the PLA group, there were 262 normal and 35 positive points, including anomia (9 points), perseveration (1 point), phonemic aphasia (6 points), repetition disorder (8 points), and semantic aphasia (11 points) (Figures 3a and S3B). There were 226 and 262 normal points in the non‐PLA and PLA groups, respectively (Figure S3). The proportion of positive responses was analyzed in each group (Figure 3b,c). In the non‐PLA group, areas with a high proportion of positive responses were found in the pSTG and pMTG. Importantly, the SMG did not show a positive response, except in one anterior location. In the PLA group, a wide distribution of high proportion of positive responses was observed, from the pSTG to the temporoparietal junction. The incidence of positive responses in the pSTG tended to be higher in the non‐PLA group (45%) than in the PLA group (26%); however, the difference was not significant (*χ* ^*2*^(1) = 1.32, *p* = .29, chi‐squared test) (Figure 3d). In contrast, the proportion of positive responses for the SMG was significantly higher in the PLA group (47%) than in the non‐PLA group (10%) (*χ* ^2^(1) = 4.36, *p* = .037, chi‐squared test) (Figure 3d). For the AG and pMTG, no significant differences were found between the groups (both *p* > .6, chi‐squared test) (Figure 3d).

The SMG was further divided into the anterior and posterior SMG (aSMG and pSMG, respectively) as previously described (Figure 3e) (Corina et al., 2005). The percentages of positive responses in the aSMG were 11% and 8% in the non‐PLA and PLA groups, respectively, with no significant difference between the groups (*χ* ^2^(1) = 0.074, *p* = .79, chi‐squared test). In contrast, the proportion of positive responses for the pSMG was significantly higher in the PLA group (47%) than in the non‐PLA group (0%) (*χ* ^2^(1) = 6.79, *p* = .0091, chi‐squared test). The tumor extended partially to the pSMG in five patients in the non‐PLA group and nine patients in the PLA group. When we excluded patients whose tumor extended to the SMG, the proportion of positive responses in the pSMG was still significantly higher in the PLA group (50%) than in the non‐PLA group (0%) (*χ* ^2^(1) = 4.19, *p* = .041) (Figure S4).

### 3.2 BC in rsfMRI connectivity

**FIGURE 4:**
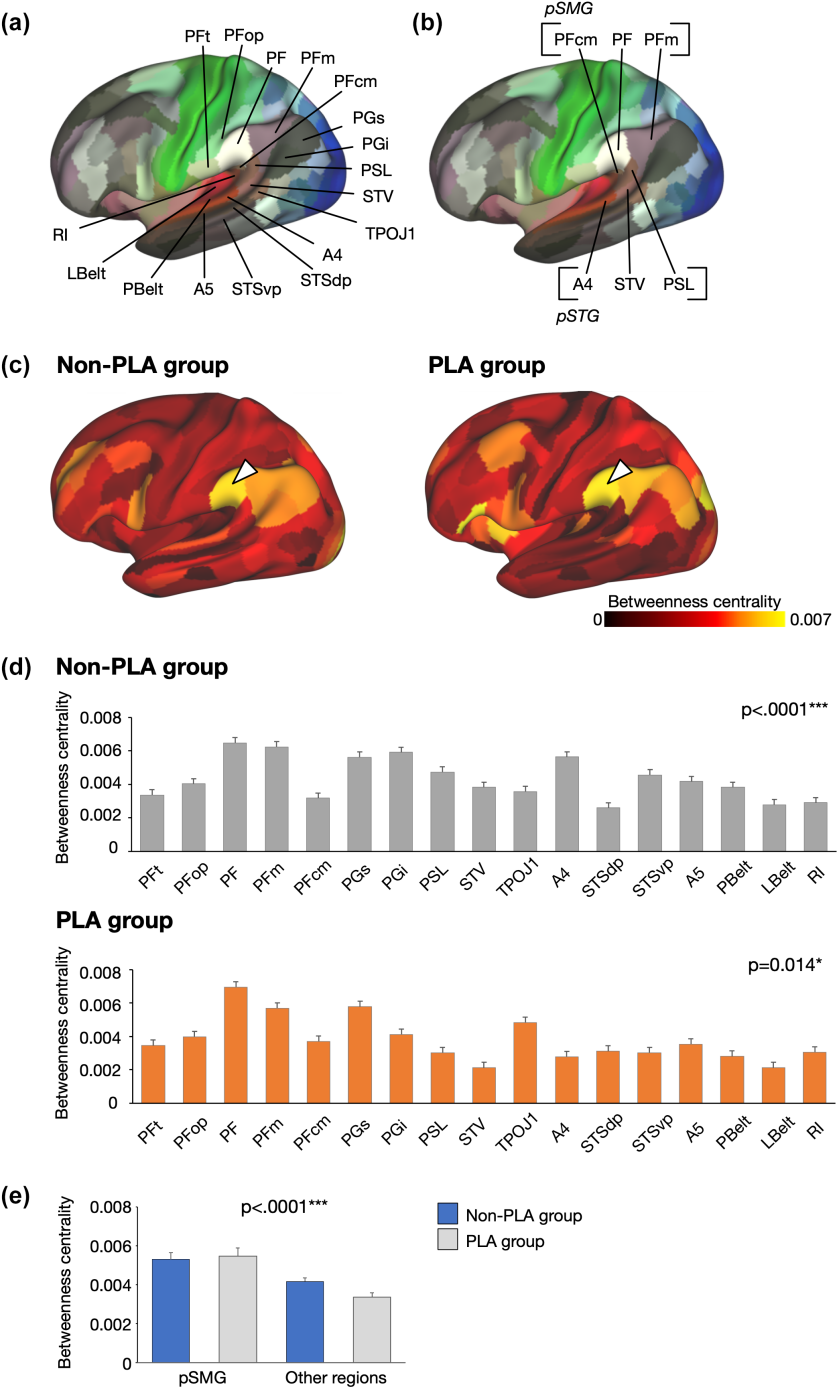
Results of resting‐state fMRI connectivity. (a) Parcels in the posterior language area (PLA) and surrounding areas are analyzed in this study. (b) The PFcm, PF, and PFm constitute the posterior part of the SMG (pSMG), and the A4, STV, and PSL constitute the posterior part of the superior temporal gyrus (pSTG). (c) Betweenness centrality (BC) in the non‐PLA and PLA groups. Higher BC is observed in the pSMG, specifically in the PF (white triangles) in both the groups. (d) The BC of the non‐PLA (gray) and PLA (orange) groups for each parcel. Analysis of variance (ANOVA) reveals significant main effects in both groups. Error bars indicate the standard error of means. (e) The BC of the supramarginal gyrus (SMG) and other surrounding regions for the non‐PLA (blue) and PLA (gray) groups. Mixed ANOVA with the areas (pSMG/other regions) and patient groups (non‐PLA/PLA groups) as the main effects revealed that the BC in the pSMG was significantly higher than that in the other surrounding regions. *p < .05; ***p < .001.

Figure 4 shows the BC in the PLA and surrounding areas in the two groups (Figure 4a), particularly in the pSTG (i.e., A4, STV, and PSL) and pSMG (i.e., PFcm, PF, and PFm) (Glasser et al., 2016) (Figure 4b). The BC in the pSMG, particularly in the PF, tended to be high in both the groups (Figure 4c). One‐way analysis of variance (ANOVA) revealed differential BC in both the non‐PLA (*F*(1,16) = 4.25, *p* = 1.07 × 10^−7^) and PLA groups (*F*(1,16) = 2.01, *p* = .014) (Figures 4d and S5A). We then performed mixed ANOVA with the areas (pSMG/other regions) and patient groups (non‐PLA/PLA groups) as the main effects. The BC in the pSMG was significantly higher than that in the other surrounding areas (*F*(1,38) = 35.2, *p* = 7.00 × 10^−7^) (Figures 4e and S5B). Other graph theoretical measures (degree centrality, page‐rank centrality, and eigenvector centrality) and binarization of resting‐state functional connectivity with other thresholds (top 5%, 15%, and 20%) also showed that the pSMG exhibited higher centrality than other areas (Figure S6A,B). Additionally, the same analysis was performed separately for WHO grades 2 and 3 versus 4. The results for grades 2 and 3 were similar to the overall results (*F*(1,24) = 30.0, *p* = 2.7 × 10^−5^), but no significant results could be found for grade 4 due to the small number of cases (*F*(1,12) = 4.54, *p* = .054) (non‐PLA group, *n* = 6 vs. PLA group, *n* = 8) (Figure S6C).

BC was calculated using another dataset of patients with tumors in the right hemisphere as an age‐matched control. The results revealed that the pSMG still exhibited higher centrality than other regions (*t*(21) = 2.88, *p* = 8.9 × 10^−3^) (Figure S6D). We also analyzed the normal control group from the HCP and found that the pSMG exhibited higher centrality than the other regions (*t*(39) = 3.05, *p* = 4.2 × 10^−3^) (Figure S6E). These findings suggest that the higher centrality in the pSMG in the left hemisphere, observed in both the PLA and non‐PLA groups, was inherent to all subjects investigated and not dependent on the presence of tumors.

## 4 DISCUSSION

In the current study, we analyzed the characteristics of reorganization in the ipsilateral hemisphere of PLA using awake brain mapping and rsfMRI. Most of the positive points in the non‐PLA group were limited to the pSTG, whereas the PLA group had positive points extending to the pSMG. Network analyses using rsfMRI further revealed that the pSMG had high BC in both the groups. Awake brain mapping and neuroimaging analyses suggest that the language area might spread to the pSMG, which can be considered a hub region, under the condition of tumor invasion to the PLA.

The positive points of all patients reported in this study were distributed in the pSTG, pMTG, pSMG, and AG. The distribution was found to be heterogeneous depending on the PLA/non‐PLA group. The positive areas, located mainly in the pSTG in the non‐PLA group, may represent the classical language area. Several fMRI studies using naming tasks in healthy individuals have demonstrated that the left pSTG activation was robust and that the SMG activation was limited (Gkiatis et al., 2022; Smitha et al., 2017; Vasileiadi et al., 2023). Therefore, we speculate that the relatively wider distribution of language areas that include the areas around the pSMG reported in previous studies on DES reflects functional reorganization in patients with tumors in the PLA (Sarubbo et al., 2020; Tate et al., 2014).

It has been reported that in several diseases, including epilepsy, the efficiency of global networks is not different between normal brains and brains with focal damage (Ridley et al., 2015). The substitution in the pSMG with high centrality can be interpreted from the following viewpoints. The pSMG is an association area involved in the integration of different sensory information and various cognitive tasks, such as different language operations (e.g., verbal working memory, phonological processing, and comprehension) (Andersen, 1997; Culham & Kanwisher, 2001; Deschamps et al., 2014). In other words, this may indicate that the parietal association cortex in the pSMG has the potential to compensate for various functions, including language. Another possibility is that the brain attempts to maintain the system‐level efficiency of neural networks by rearranging functional areas into hub regions with rich connections with other nodes (Ridley et al., 2015). Thus, it may be reasonable to compensate in the parietal association cortex to maintain the efficiency of the brain network. A previous study, which examined structural changes using longitudinal MRI data after stroke, suggests the possibility of reorganization of brain function to the hub region (Chen et al., 2021). In the study, common and unique gray matter changes in the left and right lesions overlapped with the highly connected cortical hub region in healthy participants, and these changes correlated with behavioral recovery. This suggests that the cortical hub region plays an important role in plasticity after brain damage.

There is another possible reason for the similar high BC values of the pSMG in both groups. Some patients in the non‐PLA group had lesions in the white matter tract connected to the PLA, such as the arcuate fasciculus. In a previous study, some patients with damaged nerve fibers had reduced functional connectivity in the cortex, which is the terminal region of the fiber, and functional connectivity in other regions may have increased as compensation (Matsubayashi et al., 2023). However, we also found high BC values in both the control group whose tumors were on the right side and the normal control group, which may suggest that the high BC in the pSMG was inherent but not induced by the presence of tumors. Therefore, regarding the pSMG, it is less likely that the tumors in the non‐PLA group interacted with the white matter tract connected to the PLA and induced similar changes in the SMG. It is also to be noted that glioma cells in the brain are suggested to disrupt functional connectivity measured by BOLD signal due to (i) decreased neurovascular coupling and (ii) damaged neural connections (Hadjiabadi et al., 2018; Stoecklein et al., 2020). The decreased BC in the pSTG observed in the PLA group may be attributed to the disturbed BOLD signal and/or disrupted functional connectivity resulting from the presence of glioma in the pSTG, rather than indicating a shift in functionality.

Recently, the usefulness of rsfMRI in identifying language areas before surgery has been attracting attention. It has been reported that the language networks identified through rsfMRI and DES in awake surgery agree with each other with a high degree of accuracy (Lemee et al., 2019). On the other hand, the hubs identified by graph‐theoretic analysis are not specific, and their relation to awake mapping is not known. At the individual level, surgical resection of a high centrality area could cause the neurological deficit, including declining language task performance (Lee et al., 2020). Since the hub may be changed by tumor effects (Tuovinen et al., 2016), analysis at the individual level may reveal an association between awake mapping and hub of functional connectivity.

DES data was obtained for a surgical purpose and not for a research purpose, and the spatial extent of DES data was limited to the surgical area. Ideally, to examine functional reorganization, it is more appropriate to compare the language area of patients with gliomas with that of healthy individuals or to compare the language area before and after the appearance of lesions in the same individual. Hence, in accordance with a previous study (Saito et al., 2016), this study compared the distribution of the language area between the PLA and non‐PLA groups. Considering all these facts, a longitudinal study is needed to validate whether brain function indeed moves from the pSTG to the pSMG. Another limitation is the possible sampling bias of stimulation points between the PLA and non‐PLA groups. In particular, there were a small number of positive responses around the language area in the non‐PLA group. This is due to the limited opportunities to systematically stimulate the area around the PLA in patients whose tumor was separate from the language area during clinical practice. Moreover, we cannot rule out the possibility of some spatial inaccuracies in stimulation points due to the localization procedure. Additionally, our patient group included grades 2 and 3, and grade 4. Reorganization can occur more frequently in lower‐grade gliomas than in higher‐grade gliomas. In the case of higher‐grade glioma, however, the reorganization process may still be in progress at the preoperative timepoint (Cargnelutti et al., 2020). Since peritumoral reorganization is considered an early compensatory mechanism (Nieberlein et al., 2023), reorganization should occur in high‐grade gliomas, but further studies are required to clarify the difference in reorganization patterns among malignancy grades.

## 5 CONCLUSIONS

In patients with lesion progression to the pSTG, the language area was distributed widely to the pSMG, or hub region beyond the PLA. Functional substitution in hub regions may be considered a reasonable way to maintain the efficiency of the brain network. The combined use of DES and functional connectivity allowed us to characterize the detailed change in the pattern of the language area within the ipsilateral hemisphere. The pattern of perilesional reorganization may also be applicable to other cortical areas and should be validated in a longitudinal study with a large number of cases in the future.

## FUNDING INFORMATION

This work was supported by JSPS KAKENHI (grant numbers 20K21649 and 22K18397 to M.N. and 21H03301 and 21K19705 to R.N.).

## CONFLICT OF INTEREST STATEMENT

The authors report no conflict of interest concerning the materials or methods used in this study or the findings specified in this paper.

## PATIENT CONSENT

Written informed consent was obtained from all patients.

## Supporting information


**Figure S1.** Flowchart of the inclusion criteria.

**Figure S2.** Additional information on methods used in the study. (A) The flow chart shows the number of patients included in each analysis. (B) In Figures 2b and 3b, we calculated the ratio of the number of patients with positive responses to the number of electrically stimulated patients for each square as the frequency of patients with positive responses. A square is considered a positive response if each square has at least one positive point. Even if there are two or more positive points in a square, that square is counted as only one positive response. The same is applied to normal responses. Note that the number of patients with positive responses in each square (Figures 2c and 3b) should be lesser than the number of positive points that can be counted in Figures 2a and 3a.

**Figure S3.** Normal points in the non‐PLA (A) and PLA groups (B). Since the space in the brain surface is limited and some of the points overlap, displaying all of them is difficult.

**Figure S4.** Intraoperative findings of the non‐PLA and PLA groups, excluding patients whose tumor extended to the SMG. Patients whose tumor extended to the SMG are excluded from the non‐PLA group (*n* = 5) and PLA group (*n* = 9) and re‐analyzed. (A and B) Maps of tumor overlap across patients in the non‐PLA (*n* = 37) and PLA (*n* = 11). The frequency of positive responses is calculated as the ratio (%) of the number of patients with positive responses to the number of patients stimulated. (C) The percentages of positive responses in the pSTG, SMG, AG, and pMTG in the non‐PLA and PLA groups (excluding patients in whom the tumor extended to the SMG). Red, positive response; blue, normal response. (D) The SMG is further divided into two parts, anterior and posterior SMG (aSMG and pSMG). The number under the horizontal axis in figure C and D represents the number of patients stimulated in each gyrus. *p < .05; **p < .01. PLA, posterior language area; aSMG, anterior part of the supramarginal gyrus; pSMG, posterior part of the supramarginal gyrus.

**Figure S5.** Dot plots for Figure 4d (A) and 4E (B). Red lines indicate the average. *p < .05; ***p < .001.

**Figure S6.** Additional analyses on results of rsfMRI. We performed mixed analysis of variance with the areas (pSMG/other regions) and patient groups (non‐PLA/PLA groups) as the main effects in the following conditions: different centralities (A) including degree centrality, page‐rank centrality, and eigenvector centrality; different thresholds (B) including 0.05, 0.15, and 0.20; malignancy grade (C) including grades 2 and 3, and grade 4. (D) We also analyzed betweenness centrality in the age‐matched control group of right cerebral hemispheric gliomas (*N* = 22, 45.5 ± 16.2 years) and found significant differences between areas (*t*‐test, *t*(21) = 2.88, *p* = 8.9 × 10^−3^). (E) Comparison between the areas using the normal control group from the Human Connectome Project also revealed that the pSMG exhibited higher centrality than the other regions (*t*‐test, *t*(39) = 3.05, *p* = 4.2 × 10^−3^). rsfMRI, resting‐state functional magnetic resonance imaging; pSMG, posterior part of the supramarginal gyrus; PLA, posterior language area.

**Table S1.** MNI coordinates of positive points.

## Data Availability

The data that supports the findings of this study are available on request from the corresponding author. The data are not publicly available due to privacy/ethical restrictions (containing information that could compromise the privacy of research participants).
