# Supplementary figures and images for: More widespread functionality of posterior language area in patients with brain tumors
Source: Hum Brain Mapp. 2024 Aug 1;45(11):e26801. doi: 10.1002/hbm.26801 (PMC11293139; doi:10.1002/hbm.26801)

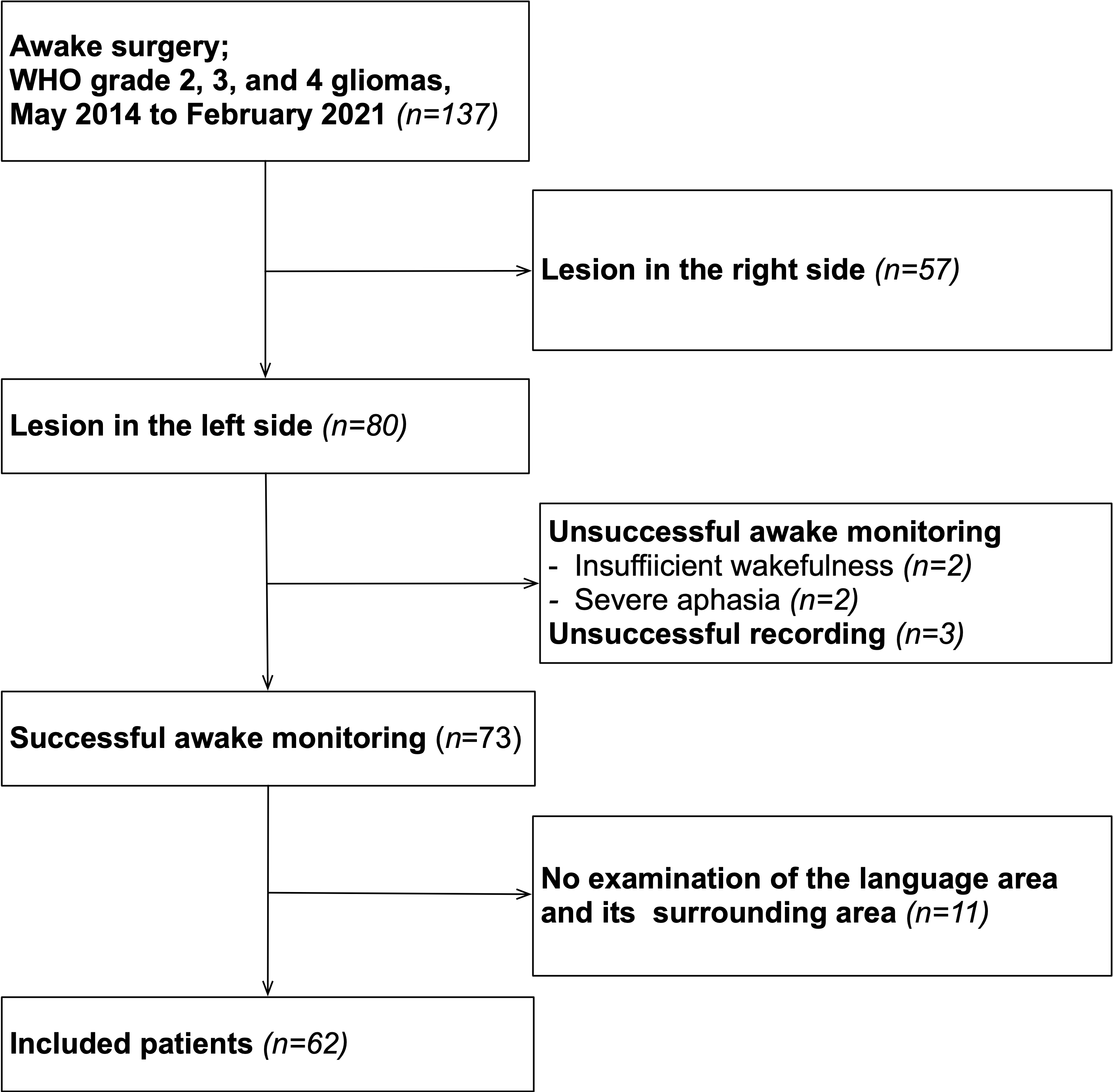

Supplement: Supplementary file 1 — Figure S1. Flowchart of the inclusion criteria. [file HBM-45-e26801-s004.tiff]

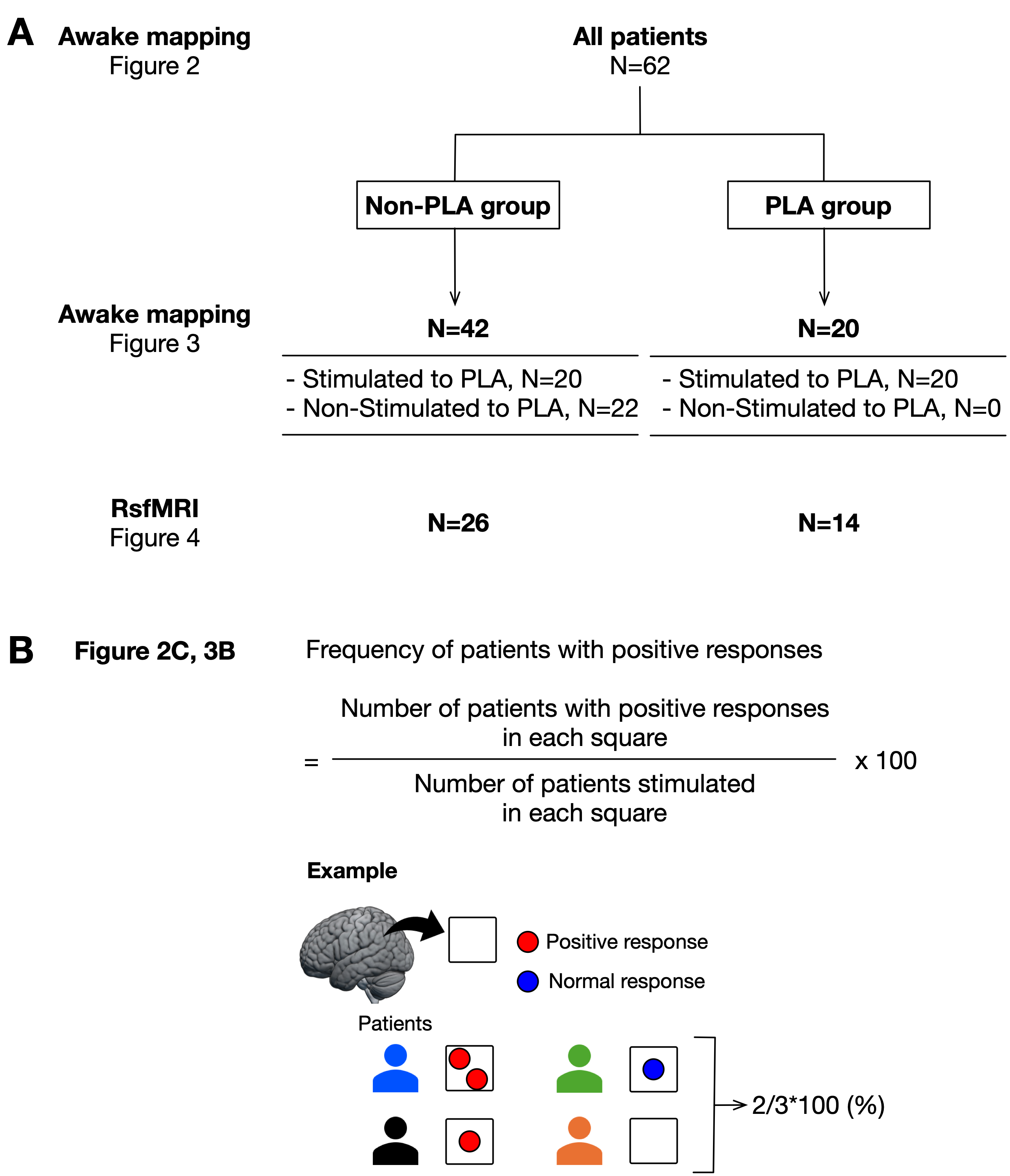

Supplement: Supplementary file 2 — Figure S2. Additional information on methods used in the study. (A) The flow chart shows the number of patients included in each analysis. (B) In Figures 2b and 3b, we calculated the ratio of the number of patients with positive responses to the number of electrically stimulated patients for each square as the frequency of patients with positive responses. A square is considered a positive response if each square has at least one positive point. Even if there are two or more positive points in a square, that square is counted as only one positive response. The same is applied to normal responses. Note that the number of patients with positive responses in each square (Figures 2c and 3b) should be lesser than the number of positive points that can be counted in Figures 2a and 3a. [file HBM-45-e26801-s005.tiff]

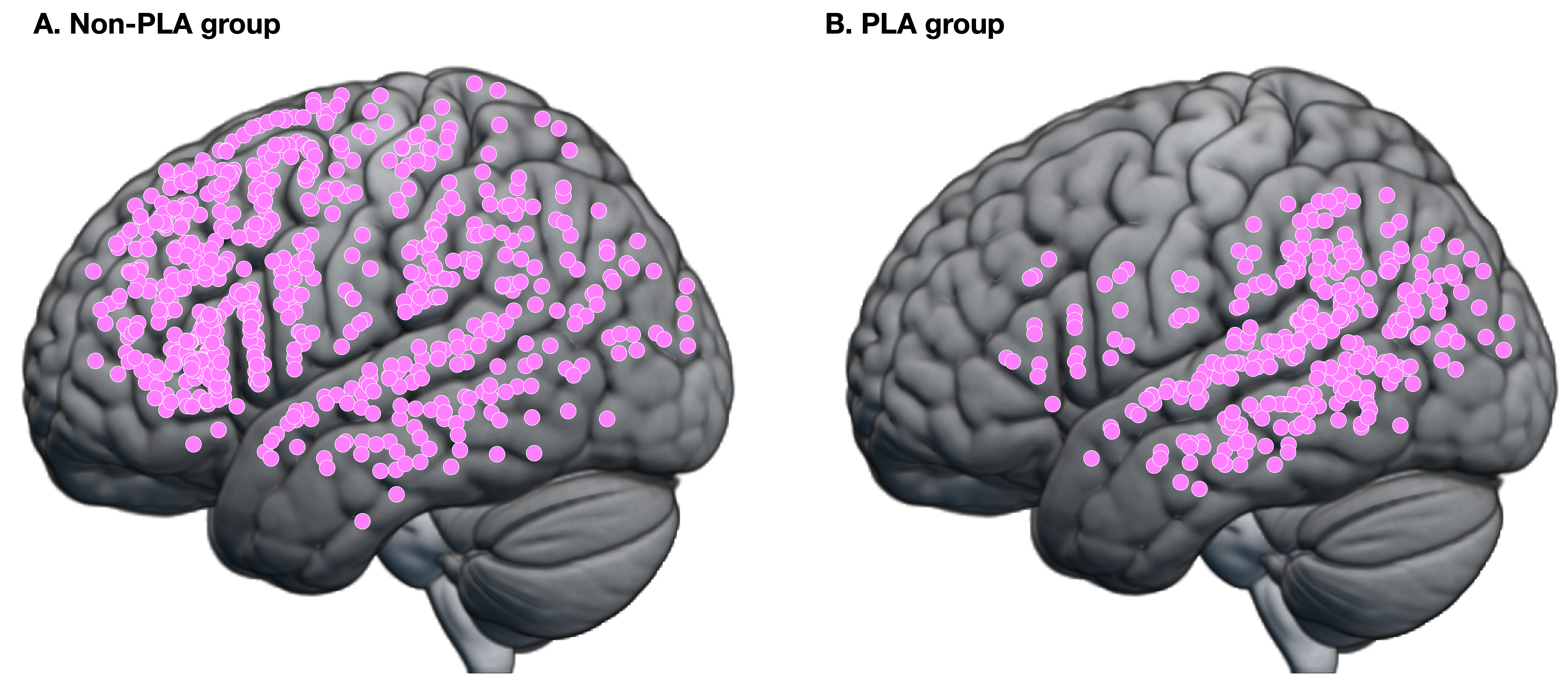

Supplement: Supplementary file 3 — Figure S3. Normal points in the non‐PLA (A) and PLA groups (B). Since the space in the brain surface is limited and some of the points overlap, displaying all of them is difficult. [file HBM-45-e26801-s001.tiff]

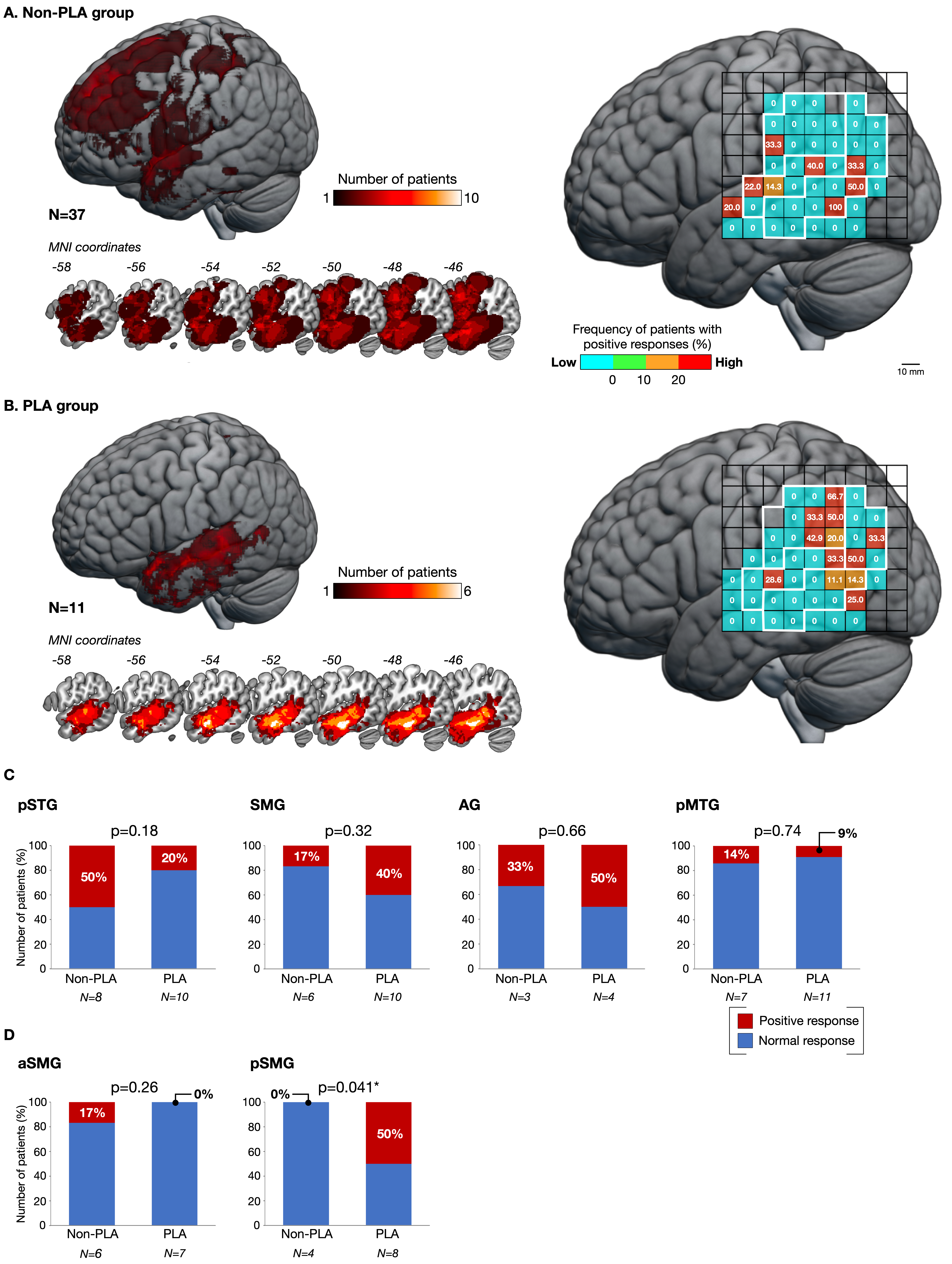

Supplement: Supplementary file 4 — Figure S4. Intraoperative findings of the non‐PLA and PLA groups, excluding patients whose tumor extended to the SMG. Patients whose tumor extended to the SMG are excluded from the non‐PLA group (n = 5) and PLA group (n = 9) and re‐analyzed. (A and B) Maps of tumor overlap across patients in the non‐PLA (n = 37) and PLA (n = 11). The frequency of positive responses is calculated as the ratio (%) of the number of patients with positive responses to the number of patients stimulated. (C) The percentages of positive responses in the pSTG, SMG, AG, and pMTG in the non‐PLA and PLA groups (excluding patients in whom the tumor extended to the SMG). Red, positive response; blue, normal response. (D) The SMG is further divided into two parts, anterior and posterior SMG (aSMG and pSMG). The number under the horizontal axis in figure C and D represents the number of patients stimulated in each gyrus. *p < .05; **p < .01. PLA, posterior language area; aSMG, anterior part of the supramarginal gyrus; pSMG, posterior part of the supramarginal gyrus. [file HBM-45-e26801-s007.tiff]

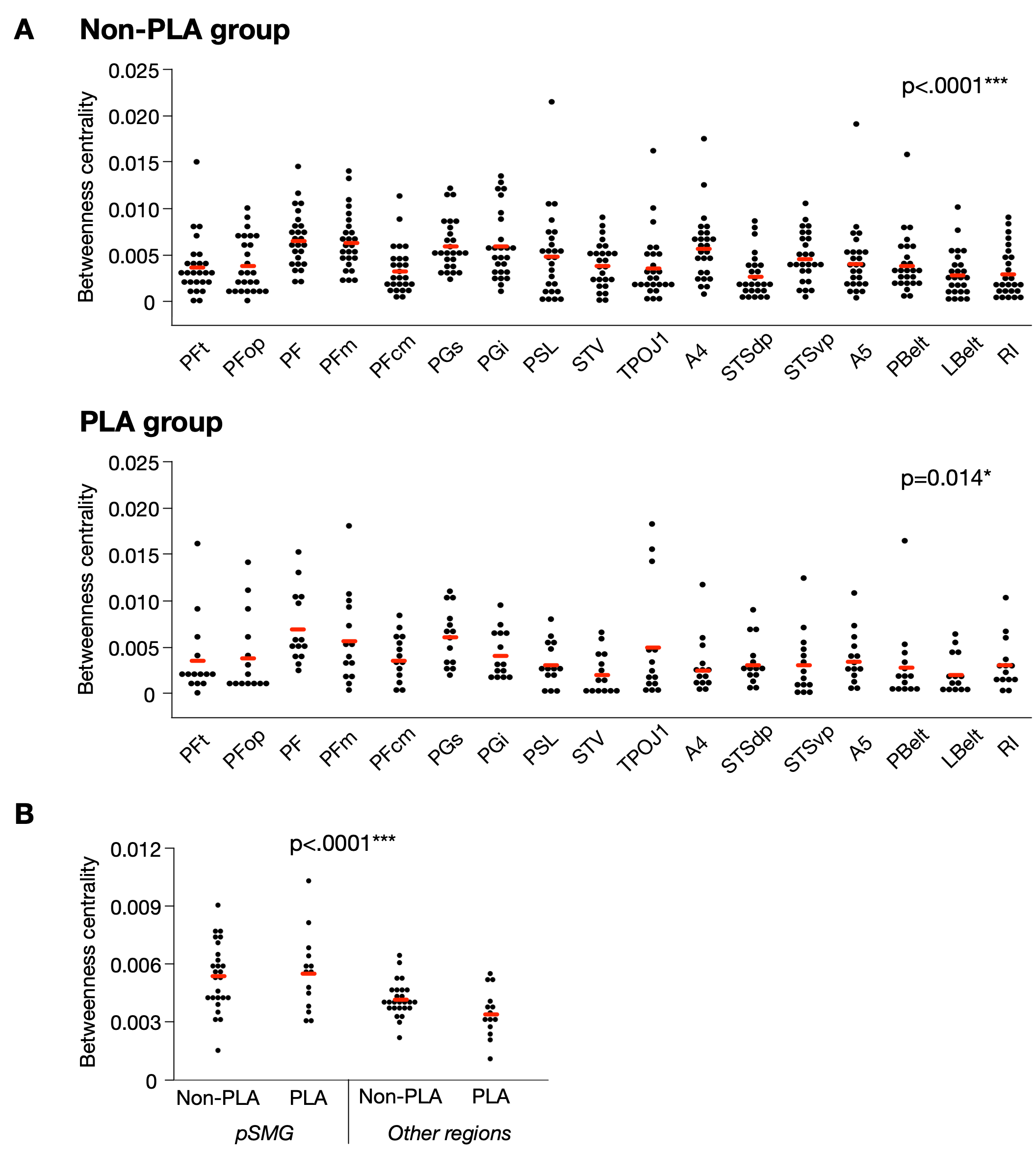

Supplement: Supplementary file 5 — Figure S5. Dot plots for Figure 4d (A) and 4E (B). Red lines indicate the average. *p < .05; ***p < .001. [file HBM-45-e26801-s003.tiff]

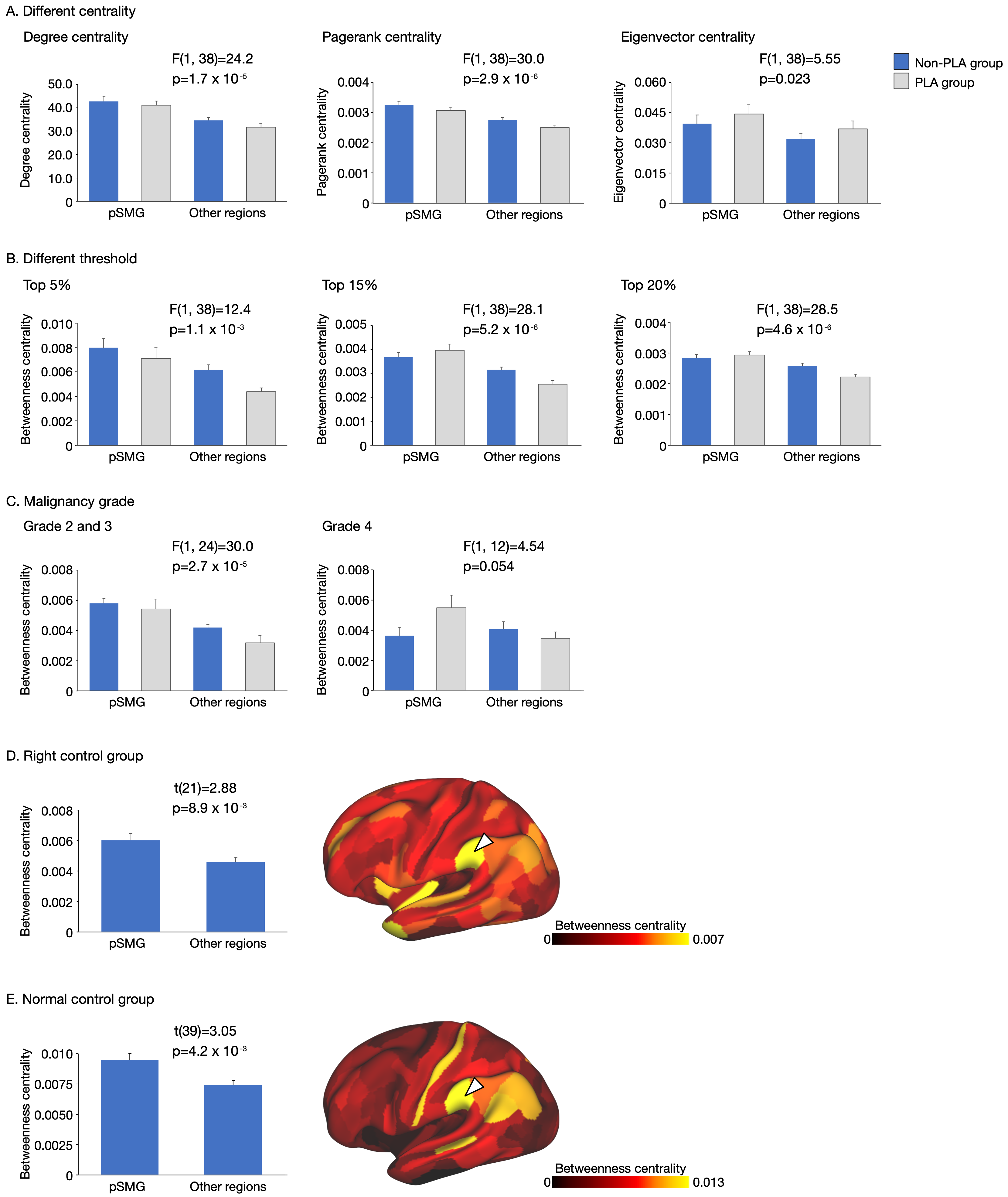

Supplement: Supplementary file 6 — Figure S6. Additional analyses on results of rsfMRI. We performed mixed analysis of variance with the areas (pSMG/other regions) and patient groups (non‐PLA/PLA groups) as the main effects in the following conditions: different centralities (A) including degree centrality, page‐rank centrality, and eigenvector centrality; different thresholds (B) including 0.05, 0.15, and 0.20; malignancy grade (C) including grades 2 and 3, and grade 4. (D) We also analyzed betweenness centrality in the age‐matched control group of right cerebral hemispheric gliomas (N = 22, 45.5 ± 16.2 years) and found significant differences between areas (t‐test, t(21) = 2.88, p = 8.9 × 10^−3^). (E) Comparison between the areas using the normal control group from the Human Connectome Project also revealed that the pSMG exhibited higher centrality than the other regions (t‐test, t(39) = 3.05, p = 4.2 × 10^−3^). rsfMRI, resting‐state functional magnetic resonance imaging; pSMG, posterior part of the supramarginal gyrus; PLA, posterior language area. [file HBM-45-e26801-s006.tiff]
