# Supplementary material for: More widespread functionality of posterior language area in patients with brain tumors
Source: Hum Brain Mapp. 2024 Aug 1;45(11):e26801. doi: 10.1002/hbm.26801 (PMC11293139; doi:10.1002/hbm.26801)
Supplement: Supplementary file 7 — Table S1. MNI coordinates of positive points. [file HBM-45-e26801-s002.docx]

**Supplementary Table 1.** MNI coordinates of positive points

| No | Responses | MNI coordinates | | |
| --- | --- | --- | --- | --- |
|  |  | X | Y | Z |
| 1 | Anomia | -63.5 | -56.5 | 7.0 |
| 2 | Anomia | -67.0 | -44.5 | 14.0 |
| 3 | Anomia | -55.5 | -54.5 | 41.5 |
| 4 | Anomia | -65.0 | -2.0 | -2.0 |
| 5 | Anomia | -59.0 | -54.5 | 29.5 |
| 6 | Anomia | -26.5 | -47.0 | 35.5 |
| 7 | Anomia | -65.5 | -37.0 | 29.5 |
| 8 | Anomia | -64.0 | -56.5 | 10.0 |
| 9 | Anomia | -60.5 | -61.0 | 6.0 |
| 10 | Anomia | -63.5 | -57.0 | 10.0 |
| 11 | Anomia | -66.5 | -50.0 | 6.0 |
| 12 | Anomia | -62.5 | -49.0 | 25.5 |
| 13 | Anomia | -68.5 | -22.0 | 8.0 |
| 14 | Anomia | -57.5 | -65.0 | 14.5 |
| 15 | Perseveration | -67.0 | -44.5 | 17.0 |
| 16 | Perseveration | -67.5 | -29.0 | 24.0 |
| 17 | Repetition | -63.0 | -50.0 | 32.0 |
| 18 | Repetition | -55.0 | -52.0 | 42.0 |
| 19 | Repetition | -61.0 | -59.5 | 22.0 |
| 20 | Repetition | -64.0 | -52.5 | 22.5 |
| 21 | Repetition | -63.5 | -57.0 | 11.5 |
| 22 | Repetition | -57.5 | -67.0 | 15.0 |
| 23 | Repetition | -57.5 | -65.5 | 4.0 |
| 24 | Repetition | -54.5 | -71.0 | 0.0 |
| 25 | Semantic | -59.0 | -65.5 | 5.5 |
| 26 | Semantic | -65.0 | -57.5 | 2.0 |
| 27 | Semantic | -67.5 | -38.0 | 14.0 |
| 28 | Semantic | -53.5 | -71.5 | 19.5 |
| 29 | Semantic | -49.5 | -60.5 | 48.5 |
| 30 | Semantic | -51.0 | -64.5 | 44.0 |
| 31 | Semantic | -67.0 | -38.0 | 16.0 |
| 32 | Semantic | -61.0 | -58.5 | 10.0 |
| 33 | Semantic | -59.5 | -65.0 | 8.0 |
| 34 | Semantic | -56.0 | -70.5 | 2.0 |
| 35 | Semantic | -65.5 | -52.0 | 20.0 |
| 36 | Semantic | -70.5 | -24.5 | 4.0 |
| 37 | Semantic | -55.5 | -70.5 | 0.5 |
| 38 | Semantic | -68.0 | -40.5 | 12.0 |
| 39 | Semantic | -60.5 | -54.5 | 30.0 |
| 40 | Phonemic | -70.0 | -44.5 | 15.5 |
| 41 | Phonemic | -58.0 | -67.0 | 14.0 |
| 42 | Phonemic | -63.5 | -52.5 | 25.0 |
| 43 | Phonemic | -67.0 | -19.0 | 3.5 |
| 44 | Phonemic | -66.5 | -10.0 | 0.0 |
| 45 | Phonemic | -70.0 | -30.0 | 7.5 |
| 46 | Phonemic | -64.5 | -49.5 | 27.0 |
| 47 | Phonemic | -54.0 | -65.5 | 36.5 |
| 48 | Phonemic | -67.0 | -41.5 | 18.0 |
